# Progressive multiple sequence alignments from triplets

**DOI:** 10.1186/1471-2105-8-254

**Published:** 2007-07-15

**Authors:** Matthias Kruspe, Peter F Stadler

**Affiliations:** 1 Bioinformatics Group, Department of Computer Science and Interdisciplinary Center for Bioinformatics, University of Leipzig, Härtelstraße 16-18, D-04107 Leipzig, Germany; 2 Fraunhofer Institut für Zelltherapie und Immunologie (IZI), Deutscher Platz 5e, D-04103 Leipzig, Germany; 3 Institute for Theoretical Chemistry, University of Vienna, Währingerstraße 17, A-1090 Wien, Austria; 4 Santa Fe Institute, 1399 Hyde Park Rd., Santa Fe, NM 87501, USA

## Abstract

**Background:**

The quality of progressive sequence alignments strongly depends on the accuracy of the individual pairwise alignment steps since gaps that are introduced at one step cannot be removed at later aggregation steps. Adjacent insertions and deletions necessarily appear in arbitrary order in pairwise alignments and hence form an unavoidable source of errors.

**Research:**

Here we present a modified variant of progressive sequence alignments that addresses both issues. Instead of pairwise alignments we use exact dynamic programming to align sequence or profile triples. This avoids a large fractions of the ambiguities arising in pairwise alignments. In the subsequent aggregation steps we follow the logic of the Neighbor-Net algorithm, which constructs a phylogenetic network by step-wisely replacing triples by pairs instead of combining pairs to singletons. To this end the three-way alignments are subdivided into two partial alignments, at which stage all-gap columns are naturally removed. This alleviates the "once a gap, always a gap" problem of progressive alignment procedures.

**Conclusion:**

The three-way Neighbor-Net based alignment program aln3nn is shown to compare favorably on both protein sequences and nucleic acids sequences to other progressive alignment tools. In the latter case one easily can include scoring terms that consider secondary structure features. Overall, the quality of resulting alignments in general exceeds that of clustalw or other multiple alignments tools even though our software does not included heuristics for context dependent (mis)match scores.

## 1 Background

(The software is freely available for download from reference [1])

High quality multiple sequence alignments (MSAs) are a prerequisite for many applications in bioinformatics, from the reconstruction of phylogenies and the assessment of evolutionary rate variations to gene finding and phylogenetic footprinting. A large part of comparative genomics thus hinges on our ability to construct accurate MSAs. Since the multiple sequence alignment problem is NP hard [2] with the computational cost growing exponentially with the number of sequences, it has been a long-standing challenge to devise approximation algorithms that are both efficient and accurate. These approaches can be classified into progressive, iterative, and stochastic alignment algorithms. The most widely used tools such as clustalw [3] and pileup utilize the progressive method that was at first introduced in [4,5]. This approach makes explicit use of the evolutionary relatedness of the sequences to build the alignment. The complete multiple sequence alignment of the given sequences is calculated from pairwise alignments of previous aligned sequences by following the branching order of a pre-computed "guide" tree, which reflects (at least approximately) the evolutionary history of the input sequences. It is typically reconstructed from pairwise sequence distances by some clustering method such as Neighbor-Joining [6] or UPGMA [7]. Progressive sequence alignments, while computationally efficient, suffer from two major shortcomings. First, they are of course not guaranteed to find the optimal alignment. Pairwise comparisons necessarily utilize only a small part of the information that is potentially available in the complete data set. In particular, the relative placement of adjacent insertions and deletions leads to score-equivalent alignments among which the algorithm chooses one by means of a pragmatic rule (e.g. "Always make insertions before deletions"). At a later aggregation step, when profiles are aligned to sequences or with each other, these alternative are no longer equivalent. Secondly, in contrast to other techniques, there is no mechanism to identify errors that have been made in previous steps and to correct them during later stages.

In this contribution we present a novel approach to progressive sequence alignment that alleviates both shortcomings at the expense of utilizing an exact algorithm to compute alignment of sequence and profile triples. Instead of using a single guide tree, we follow here the logic of phylogenetic networks as constructed by the Neighbor-Net algorithm [8] which calls for an aggregation step that constructs pairs from triples. As this requires us to subdivide 3-way alignments into pairs of alignments, it provides a chance for the removal of erroneously inserted gaps at later aggregation steps.

The contribution is organized as follows: In the following section we outline the algorithms aspects of our approach. Furthermore we describe a straightforward way of incorporating RNA secondary information. Section 3 summarizes benchmark data in comparison to other multiple alignment tools. We conclude with a brief discussion of future improvements.

## 2 Methods

### 2.1 Dynamic Programming

The basic dynamic programming scheme for pairwise sequence comparison, known as the Needleman-Wunsch algorithm [9] requires quadratic space and time. It easily translates to a cubic space and time algorithms for three sequences. Biologically plausible sequence alignment, however, require the use of non-trivial gap cost functions. While cubic time algorithms are available for arbitrary gap costs [10], affine gap costs (with a much higher penalty for opening a new gap than for extending an existing one) in general yield good results already. In this contribution we therefore use an affine gap cost model. Gotoh's algorithm solves this problem with quadratic CPU and memory requirements for two sequences [11]. The same author also described a dynamic programming scheme for the alignment of three sequences with affine gap costs [12] that requires O
 MathType@MTEF@5@5@+=feaafiart1ev1aaatCvAUfKttLearuWrP9MDH5MBPbIqV92AaeXatLxBI9gBamrtHrhAL1wy0L2yHvtyaeHbnfgDOvwBHrxAJfwnaebbnrfifHhDYfgasaacH8akY=wiFfYdH8Gipec8Eeeu0xXdbba9frFj0=OqFfea0dXdd9vqai=hGuQ8kuc9pgc9s8qqaq=dirpe0xb9q8qiLsFr0=vr0=vr0dc8meaabaqaciaacaGaaeqabaWaaeGaeaaakeaaimaacqWFoe=taaa@383D@(*n*^3^) time and space, which we use here with minor modifications.

**Figure 1:**
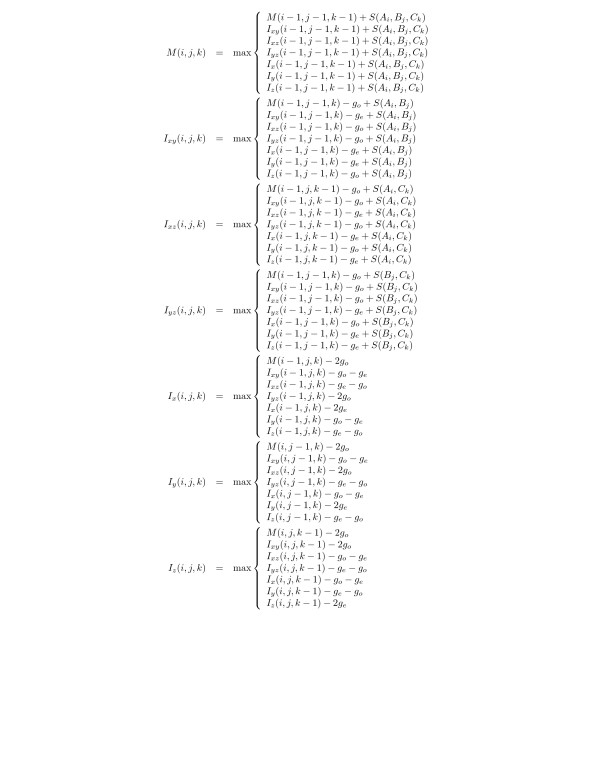
Dynamic programming recursions for three-way alignments with affine gap costs. The empty alignments are initialized as *M*(0, 0, 0) = 0 and *I*_.._(0,0,0) = 0. The boundaries of the cubic tables are initialized using the recursions above with the understanding that alternatives with negative indices are ignored.

Let *A*, *B*, and *C *denote the three sequences. We use *A*_*i*_, *B*_*j*_, and *C*_*k *_to refer to the *i*th, *j*th, and *k*th position in *A*, *B*, and *C*, respectively, counting from 1. As usual, '-' denotes the gap character. Scores for the alignment of two or three non-gap characters are denoted by *S*(*α*, *β*) and *S*(*α*, *β*, *γ*), resp. Gap penalties are determined from gap open (*g*_*o*_) and gap extensions (*g*_*e*_) scores. The best score of the alignments of the prefixes *A*_*i*_, *B*_*j*_, and *C*_*k *_is denoted by *M*(*i*, *j*, *k*) if the residues (*A*_*i*_*, B*_*j*_, *C*_*k*_) are aligned; *I*_*xy*_(*i*, *j*, *k*) the best score given that (*A*_*i*_*, B*_*j*_,-) is the last column of the partial alignment, and *I*_*x*_(*i*, *j*, *k*) the best score given that the last column is of the form (*A*_*i*_, -, -). *I*_*xz*_(*i*, *j*, *k*), *I*_*yz*_(*i*, *j*, *k*), *I*_*y*_(*i*, *j*, *k*), and *I*_*z*_(*i*, *j*, *k*) are defined analogously. It is not hard to verify that these quantities must satisfy the recursions summarized in Fig. 1. While the algorithm would in principle allow us to use arbitrary three residue substitution scores *S*(*a*, *b*, *c*) as described by [13], we restrict ourselves to the sum-of-pairs model *S*(*a*, *b*, *c*) = *S*(*a*, *b*) + *S*(*a*, *c*) + *S*(*b*, *c*).

As in the case of pairwise sequence alignments, the recursions immediately generalize to alignments of profiles so that a single sequence becomes a special case of a profile. Match and gap scores are simply added up over all triples of sequences, one from each profile.

The resource requirements of this algorithm, in particular the cubic memory consumption, are acceptable only for relative small sequence lengths *n *even on modern workstations. Several approaches have been explored in the past to reduce the search space so that long sequences can be dealt with, see e.g. [14-16].

We utilized here the Divide-&-Conquer approach described by [17] to limit both space and time requirements. Input sequences that exceed a given threshold length *l *are subsequently subdivided into smaller sequences until the length criterion is fulfilled. The partial sequences are aligned separately and the emerging alignments are concatenated afterward. The result is an approximate solution of the global multiple sequence alignment problem. The choice of the threshold length depends on sequence properties and the available amount of memory and CPU resources. For the following simulations we have chosen a length of *l *= 150. The methods described by [14,15] are known to produce optimal alignments but are much harder to implement.

### 2.2 Alignment order

The order in which sequences and profiles are aligned has an important influence on the performance of progressive alignment algorithms. In programs that are based on pairwise alignments such as clustalw or pileup, binary guide trees, which encapsulate at least an approximation to the phylogenetic relationships of the input sequences, are used to determine the alignment order. The input sequences form the leaves of this tree; each interior node corresponds to an alignment, so that the root of the guide tree represents the desired multiple alignment of all input sequences.

**Figure 2:**
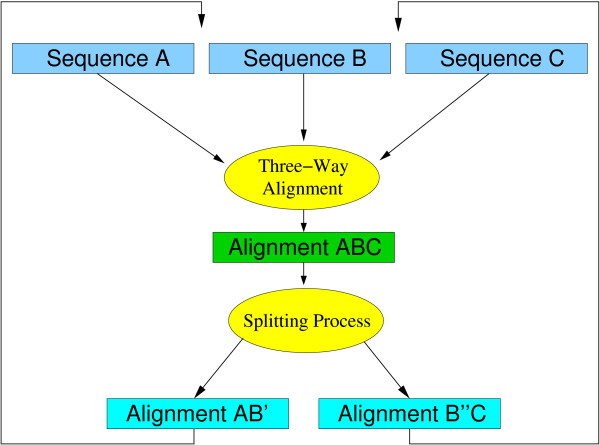
The three sequences/alignments *A*, *B*, and *C *are aligned simultaneously resulting in the alignment *ABC*. This alignment is divided into the two new alignments *AB *and *BC*. Duplicated sequences in *B *are deleted. The process continues until all sequences or alignments are aligned.

Instead of a phylogenetic tree aln3nn uses a phylogenetic network to calculate the alignment order. The network is constructed using the Neighbor-Net (Nnet) approach, a distance based clustering algorithm that can be seen as a proper generalization of Neighbor-Joining [8,18]. The Nnet algorithm can be described as follows: The input sequences are represented as nodes that are all disconnected in the beginning. In each aggregation step, Nnet selects two nodes using a specific selection criterion such as minimal distance. In contrast to Neighbor-Joining, the two nodes are not paired immediately. Instead, Nnet waits until a node has been paired up a second time. Then the corresponding three linked nodes are replaced by two new linked nodes. As in the more familiar NJ algorithm, the distances of the newly introduced nodes to the remaining "actives" node are computed as a linear combination of the distances of the nodes prior to aggregation. The entire procedure is repeated until only three active nodes are left. Then the agglomerated nodes are expanded to produce the planar splits graph that represents the desired phylogenetic network. The aggregation procedure of the Nnet algorithm *implicitly *defines a circular split system, which can be shown to be consistent in the sense that for any distance matrix that is a linear combination of split metrics deriving from a circular split system, Nnet recovers the original circular split system, see [19] for the mathematical details. It has been observed that phylogenetic distance data are often circular or at most mildly non-circular, see e.g. [20-22]. In other words, this class of phylogenetic networks very well represents distance data that obtained from pairwise sequence alignments. In our picture, each node agglomeration corresponds to a triplet alignment. The alignment order is therefore given by Nnet's order of node fusions. Nnet however replaces a triple by a pair. This suggests to split the three-way alignment again into a pair of alignments, see Fig. 2. In Nnet, a node agglomeration occurs when one of the three involved nodes (*B*) has two neighbors, while the other two (*A *and *C*) have only a single one. Following this rule, we choose to split the alignment *ABC *such the sequences contained in *B *are distributed between two subsets *B' *and *B" *so as to maximize the scores of partial alignments *AB' *and *B''C*. In practice, we start with partial alignments *AB *and *BC *obtained from *ABC*. Then each of the duplicated *B *sequences is removed from either *AB *or *BC *using a greedy rule, i.e., we remove the copy that yields the smaller average score contribution. Of course, other division strategies are conceivable. For example, one could subdivide the alignment along the longest internal edge of its Neighbor-Joining tree, or along non-trivial splits that are optimal according to other criteria. At this stage, one can either approximate the profile distances to all other intermediate alignments using Nnet's distance recursions (as implemented in nn3aln), or one could recompute these distances based on the alignments.

The division of the *ABC *alignment into *AB' *and *B''C *frequently results in all-gap columns in the two parts. These are removed in order to recover valid MSAs. This constitutes a mechanism by which gaps introduced in early agglomeration steps can be removed again in later steps. This removal is guided by the increasing amount of information that is implicit in profiles composed of a larger number of sequences. Our software keeps track of gaps that appear in intermediate alignments but that are not present in the final result to demonstrate that gap removal is not a rare phenomenon in practice.

### 2.3 Complexity

**Figure 3:**
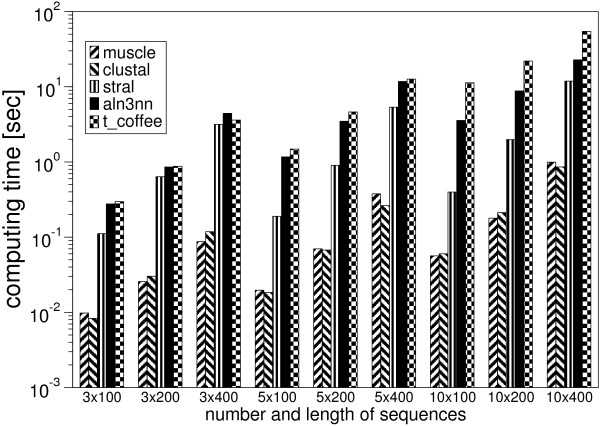
Running time in seconds for calculation of one alignment consisting of various sequences of different length on a semi-logarithmic plot for muscle, clustalw, stral, aln3nn, and t_coffee. The alignment programms are invoked all with their standard parameter settings.

The dynamic programming algorithm for the three-way alignment requires O
 MathType@MTEF@5@5@+=feaafiart1ev1aaatCvAUfKttLearuWrP9MDH5MBPbIqV92AaeXatLxBI9gBamrtHrhAL1wy0L2yHvtyaeHbnfgDOvwBHrxAJfwnaebbnrfifHhDYfgasaacH8akY=wiFfYdH8Gipec8Eeeu0xXdbba9frFj0=OqFfea0dXdd9vqai=hGuQ8kuc9pgc9s8qqaq=dirpe0xb9q8qiLsFr0=vr0=vr0dc8meaabaqaciaacaGaaeqabaWaaeGaeaaakeaaimaacqWFoe=taaa@383D@(*n*^3^) space and time (where *n *is length of the input sequences). Thus the alignment of all *N *sequences takes O
 MathType@MTEF@5@5@+=feaafiart1ev1aaatCvAUfKttLearuWrP9MDH5MBPbIqV92AaeXatLxBI9gBamrtHrhAL1wy0L2yHvtyaeHbnfgDOvwBHrxAJfwnaebbnrfifHhDYfgasaacH8akY=wiFfYdH8Gipec8Eeeu0xXdbba9frFj0=OqFfea0dXdd9vqai=hGuQ8kuc9pgc9s8qqaq=dirpe0xb9q8qiLsFr0=vr0=vr0dc8meaabaqaciaacaGaaeqabaWaaeGaeaaakeaaimaacqWFoe=taaa@383D@(*Nn*^3^) time. If the Divide-&-Conquer approach with the cutoff length *l *is used, the complexity of the alignment of one triplet can be reduced to O
 MathType@MTEF@5@5@+=feaafiart1ev1aaatCvAUfKttLearuWrP9MDH5MBPbIqV92AaeXatLxBI9gBamrtHrhAL1wy0L2yHvtyaeHbnfgDOvwBHrxAJfwnaebbnrfifHhDYfgasaacH8akY=wiFfYdH8Gipec8Eeeu0xXdbba9frFj0=OqFfea0dXdd9vqai=hGuQ8kuc9pgc9s8qqaq=dirpe0xb9q8qiLsFr0=vr0=vr0dc8meaabaqaciaacaGaaeqabaWaaeGaeaaakeaaimaacqWFoe=taaa@383D@(*n*^2 ^+ *l*^3^) space. This is the space needed to store the additional-cost matrices (see [17]) plus the space required for aligning the remaining (sub)sequences of length at most *l*. The time complexity is given by O
 MathType@MTEF@5@5@+=feaafiart1ev1aaatCvAUfKttLearuWrP9MDH5MBPbIqV92AaeXatLxBI9gBamrtHrhAL1wy0L2yHvtyaeHbnfgDOvwBHrxAJfwnaebbnrfifHhDYfgasaacH8akY=wiFfYdH8Gipec8Eeeu0xXdbba9frFj0=OqFfea0dXdd9vqai=hGuQ8kuc9pgc9s8qqaq=dirpe0xb9q8qiLsFr0=vr0=vr0dc8meaabaqaciaacaGaaeqabaWaaeGaeaaakeaaimaacqWFoe=taaa@383D@(*n*^2 ^+ *nl*^2^). The term *n*^2 ^results from the time that is needed to calculate the additional cost matrices plus the time to search for the optimal slicing positions. The term *nl*^2 ^comes from the alignment of the triplet itself. We assume for simplicity that all sequences have the same length *n *= *l*·2^*D *^(*D *= 1, 2, ... is the number of dividing levels) and all slicing positions are located exactly at the midpoint of the (sub)sequences. The total time complexity of the alignment is therefore O
 MathType@MTEF@5@5@+=feaafiart1ev1aaatCvAUfKttLearuWrP9MDH5MBPbIqV92AaeXatLxBI9gBamrtHrhAL1wy0L2yHvtyaeHbnfgDOvwBHrxAJfwnaebbnrfifHhDYfgasaacH8akY=wiFfYdH8Gipec8Eeeu0xXdbba9frFj0=OqFfea0dXdd9vqai=hGuQ8kuc9pgc9s8qqaq=dirpe0xb9q8qiLsFr0=vr0=vr0dc8meaabaqaciaacaGaaeqabaWaaeGaeaaakeaaimaacqWFoe=taaa@383D@(*Nn*^2 ^+ *Nnl*^2^). The determination of the alignment order runs in O
 MathType@MTEF@5@5@+=feaafiart1ev1aaatCvAUfKttLearuWrP9MDH5MBPbIqV92AaeXatLxBI9gBamrtHrhAL1wy0L2yHvtyaeHbnfgDOvwBHrxAJfwnaebbnrfifHhDYfgasaacH8akY=wiFfYdH8Gipec8Eeeu0xXdbba9frFj0=OqFfea0dXdd9vqai=hGuQ8kuc9pgc9s8qqaq=dirpe0xb9q8qiLsFr0=vr0=vr0dc8meaabaqaciaacaGaaeqabaWaaeGaeaaakeaaimaacqWFoe=taaa@383D@(*N*^3^) time and O
 MathType@MTEF@5@5@+=feaafiart1ev1aaatCvAUfKttLearuWrP9MDH5MBPbIqV92AaeXatLxBI9gBamrtHrhAL1wy0L2yHvtyaeHbnfgDOvwBHrxAJfwnaebbnrfifHhDYfgasaacH8akY=wiFfYdH8Gipec8Eeeu0xXdbba9frFj0=OqFfea0dXdd9vqai=hGuQ8kuc9pgc9s8qqaq=dirpe0xb9q8qiLsFr0=vr0=vr0dc8meaabaqaciaacaGaaeqabaWaaeGaeaaakeaaimaacqWFoe=taaa@383D@(*N*) space. The calculation of the necessary pairwise distances takes O
 MathType@MTEF@5@5@+=feaafiart1ev1aaatCvAUfKttLearuWrP9MDH5MBPbIqV92AaeXatLxBI9gBamrtHrhAL1wy0L2yHvtyaeHbnfgDOvwBHrxAJfwnaebbnrfifHhDYfgasaacH8akY=wiFfYdH8Gipec8Eeeu0xXdbba9frFj0=OqFfea0dXdd9vqai=hGuQ8kuc9pgc9s8qqaq=dirpe0xb9q8qiLsFr0=vr0=vr0dc8meaabaqaciaacaGaaeqabaWaaeGaeaaakeaaimaacqWFoe=taaa@383D@(*N*^2^*n*^2^) time and O
 MathType@MTEF@5@5@+=feaafiart1ev1aaatCvAUfKttLearuWrP9MDH5MBPbIqV92AaeXatLxBI9gBamrtHrhAL1wy0L2yHvtyaeHbnfgDOvwBHrxAJfwnaebbnrfifHhDYfgasaacH8akY=wiFfYdH8Gipec8Eeeu0xXdbba9frFj0=OqFfea0dXdd9vqai=hGuQ8kuc9pgc9s8qqaq=dirpe0xb9q8qiLsFr0=vr0=vr0dc8meaabaqaciaacaGaaeqabaWaaeGaeaaakeaaimaacqWFoe=taaa@383D@(*n*^2^) space. Typical running times for various sets of alignments with different numbers and lengths of sequences are shown in Figure 3. These are taken on an Intel P4 3.0 GHz equipped with 2 GB RAM running Fedora Core 5. The full source code of the program package is available free for academic users. The code will compile and run well on any machine with a full ANSI conforming C compiler and an installed Vienna RNA package [23,24] for the RNA specific scoring function. The Vienna RNA package can be obtained from [25]. The aln3nn source code and documentation is available from [1].

### 2.4 Alignments of Structured RNAs

Recent discoveries of a large number of small RNAs with distinctive secondary structures has prompted the development of specialized multiple alignment programs for this class of molecules. Most of these approaches make explicit use of structural alignment techniques such as tree editing (MARNA [26]), tree alignments (RNAforester [27]), or variants of the Sankoff algorithm [28] (foldalign [29], dynalign [30], locarna [31]). In contrast, "structure enhanced" approaches utilize standard sequence alignment algorithms but incorporate modified match and mismatch scores designed to take structural information into account [32]. The STRAL program [33] recently has demonstrated that such "structure enhanced" alignments perform comparable to true structural alignments in many cases. We have thus included in our software the possibility to use RNA secondary structure annotation as additional input with nucleic acid alignments.

We use McCaskill's algorithm [34] (as implemented in the Vienna RNA package) to compute the matrix of equilibrium base pairing probabilities *P*_*ij *_for each input sequence and derive for each sequence position the probabilities *p*^1^(*i*) = ∑_*j*<*i *_*P*_*ij*_, *p*^2^(*i*) = ∑_*j*>*i *_*P*_*ij*_, and *p*^3^(*i*) = 1 - *p*^1^(*i*) - *p*^2^(*i*) that sequence position *i *is paired with a position *j *<*i*, a position *j *> *i*, or that it remains unpaired, resp. The *p*^*x*^(*i*)-values are used as structure annotation. For a pair of annotated input sequences *A *and *B *we define structural score contributions for positions *i *and *j *by Sstruct(iA,jB)=p1(iA)⋅p1(jB)+p2(iA)⋅p2(jB)+p3(iA)⋅p3(jB)
 MathType@MTEF@5@5@+=feaafiart1ev1aaatCvAUfKttLearuWrP9MDH5MBPbIqV92AaeXatLxBI9gBaebbnrfifHhDYfgasaacH8akY=wiFfYdH8Gipec8Eeeu0xXdbba9frFj0=OqFfea0dXdd9vqai=hGuQ8kuc9pgc9s8qqaq=dirpe0xb9q8qiLsFr0=vr0=vr0dc8meaabaqaciaacaGaaeqabaqabeGadaaakeaacqWGtbWudaWgaaWcbaGaee4CamNaeeiDaqNaeeOCaiNaeeyDauNaee4yamMaeeiDaqhabeaakiabcIcaOiabdMgaPnaaBaaaleaacqWGbbqqaeqaaOGaeiilaWIaemOAaO2aaSbaaSqaaiabdkeacbqabaGccqGGPaqkcqGH9aqpdaGcaaqaaiabdchaWnaaCaaaleqabaGaeGymaedaaOGaeiikaGIaemyAaK2aaSbaaSqaaiabdgeabbqabaGccqGGPaqkcqGHflY1cqWGWbaCdaahaaWcbeqaaiabigdaXaaakiabcIcaOiabdQgaQnaaBaaaleaacqWGcbGqaeqaaOGaeiykaKcaleqaaOGaey4kaSYaaOaaaeaacqWGWbaCdaahaaWcbeqaaiabikdaYaaakiabcIcaOiabdMgaPnaaBaaaleaacqWGbbqqaeqaaOGaeiykaKIaeyyXICTaemiCaa3aaWbaaSqabeaacqaIYaGmaaGccqGGOaakcqWGQbGAdaWgaaWcbaGaemOqaieabeaakiabcMcaPaWcbeaakiabgUcaRmaakaaabaGaemiCaa3aaWbaaSqabeaacqaIZaWmaaGccqGGOaakcqWGPbqAdaWgaaWcbaGaemyqaeeabeaakiabcMcaPiabgwSixlabdchaWnaaCaaaleqabaGaeG4mamdaaOGaeiikaGIaemOAaO2aaSbaaSqaaiabdkeacbqabaGccqGGPaqkaSqabaaaaa@71AC@ This rewards bases that share similar structural properties. The total (mis)match score is the weighted sum of the sequence score and the structure score using the equation *S*_final_(*i*_*A*_, *j*_*B*_) = *ψ*·*S*_seq_(*i*_*A*_, *j*_*B*_) + (1 - *ψ*)·*S*_struct_(*i*_*A*_,*j*_*B*_) with a balance term *ψ *that measure the relative contribution of sequence and structure similarity. In the case of very similar sequence one should use *ψ *≈ 1 since inaccuracies in the structure prediction are more harmful than the extra information in this case. Conversely, very dissimilar sequences have to be aligned with a score dominated by the structural component.

## 3 Results

### 3.1 Pairwise versus Three-Way Alignments

**Figure 4:**
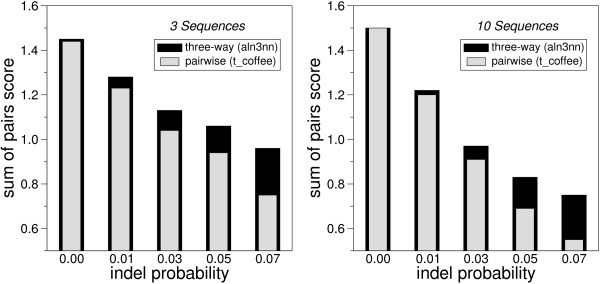
Comparison of alignments scores of aln3nn with progressive pairwise alignments for simulated data sets for different in/del rates. Data are averages over 100 simulated sets of 3 and 10 related nucleotide sequences, resp., with an average length of 100 nt. The sequences in each set are derived using ROSE from a randomly generated root sequence following the order of a given phylogenetic tree with randomly chosen branch lengths using a constant mean substitution frequency of 0.13 across the dataset. The following scoring model was used: Match score 1.9, mismatch 0.0 (as in the IUB DNA scoring matrix), gap open 2.0, gap extensions 0.5).

In order to test whether the additional computational costs of explicit three-way alignments is worth while, we generated sets of artificial sequences using the ROSE package [35] and compared the quality of aln3nn alignments to standard progressive alignments of three sequences using t_coffee. To this end we used the same scoring model in aln3nn and t_coffee so that the resulting scores can be compared directly. We report the main pairwise alignment score divided by the length of the alignment as "*pw-score*". Figure 4 shows that, as expected, the alignment score decrease quickly with increasing in/del probabilities. At the same time, the advantage of the three-way alignments increases both for the alignment of three and ten sequences. In the case of three sequences, aln3nn computes the exact solution, while t_coffee uses multiple pairwise alignments to include more information than a simple pairwise progressive alignment by modifying the pairwise scoring functions base of the consistency of a collection of pairwise alignments. Clearly, these heuristics cannot fully compensate for shortcomings of the initial pairwise alignments. The inclusion of more pairwise alignments in t_coffee heuristic does not seem to have a strong effect, at least on artifically generated sequences.

Somewhat surprisingly, three-way alignments also provide a small but significant gain in alignment score even in the cases where the simulated data correspond to a correct alignment that is entirely gap free. This effect is noticable in particular in comparison with "straight" pairwise progressive clustalw alignments, in which no attempt is made to correct for problems in the initial pairwise alignments (Additional file 1). This introduction of spurious gaps is well-known problem with pairwise nucleic acid alignments.

### 3.2 Protein Alignments

**Figure 5:**
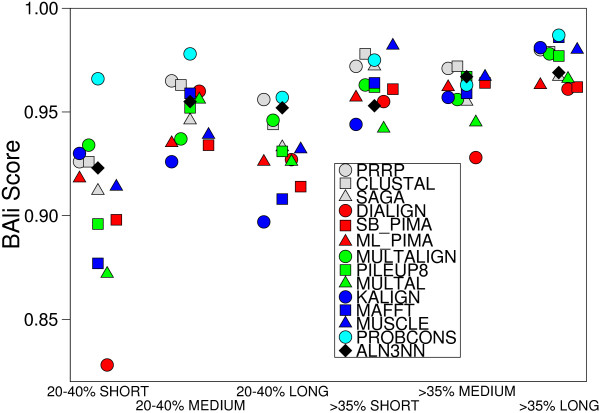
Comparison of different alignment programs on several BAliBASE benchmark data sets. Shown is an example of the Reference 1 set for mean sequence identities of 20 – 40% as well as > 35% encompassing short, medium and long sequences. Other datasets show similar results.

The aln3nn software is designed for the alignment of both amino acid and nucleic acid sequences. For proteins, the current implementation used three types of substitution matrices: BLOSUM, PAM and GONNET. The algorithm chooses the best suiting matrix of the given type according to sequence identity. The user can also specify a certain substitution matrix explicitly. We used benchmark data sets and alignments of various alignments tools from BAliBASE [36] to asses the quality of the aln3nn alignments. To assure statistical robustness, we utilized the median BAliBASE score for each sequence set as a measurement for alignment quality, Figure 5.

Our software does not employ any heuristic rules to alter scoring parameters based on local sequence context or properties of partial profiles. Nevertheless, aln3nn compares well with other common alignment programs, indicating that a simple affine scoring model is sufficient; only ProbCons [37], a combination of probabilistic modeling and consistency-based alignment techniques specialized for protein alignments performs systematically better. Elaborate scoring heuristics thus essentially seem to compensate for the algorithmic shortcomings of MSAs based on initial pairwise alignments.

### 3.3 RNA alignments

**Figure 6:**
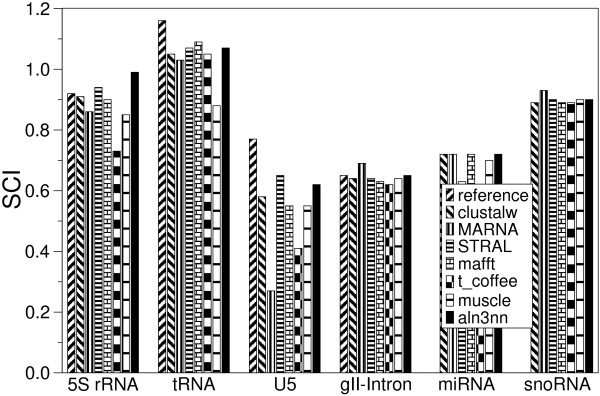
Comparison of alignment accuracies of various multiple sequence alignment tools on BRaliBase test sets. The median structure conservation index, SCI, is shown for Group II introns, 5S rRNA, tRNA, and U5 spliceosomal RNA. The relative weight of sequence and structure scores is set to *ψ *= 0.5 for all data sets.

**Figure 7:**
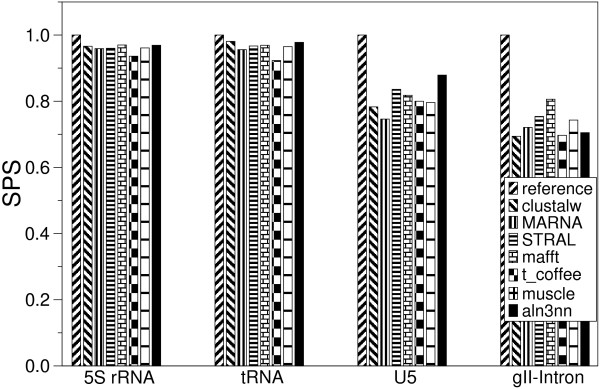
Comparison of alignment accuracies of various multiple sequence alignment tools on BRaliBase test sets. Here the median BAliBase SP score, SPS, is shown for Group II introns, 5S rRNA, tRNA, and U5 spliceosomal RNA. For the miRNA and snoRNA data set no reference alignments are available in BRaliBase.

RNA sequences often evolve much faster than their secondary structure. This is true in particular for many of the non-coding RNA genes, including ribosomal RNAs, tRNAs, and spliceosomal RNAs. In these cases, alignment quality can be increased dramatically by including structural information. In Fig. 6 and 7 we compare structure enhanced aln3nn alignments with pure sequence alignments (clustalw, muscle [38], t_coffee [39], pair-wise structure enhanced alignments (STRAL, mafft [40]) and true structural alignments (MARNA [26]) as well as the manually curated reference alignments for Rfam (v.5.0) [41]. We use six diverse families of RNA data sets from the BRaliBase that have been used in an extensive benchmark study of RNA multiple alignment algorithms [42]: Group II introns, 5S rRNA, tRNA, and U5 spliceosomal RNA. In addition, we use the data sets compiled by Jana Hertel for training SVMs that recognize microRNAs [43] and snoRNAs [44]. For each family we selected approximately 100 alignments, each consisting of five sequences encompassing a range of sequence distances.

As in [42], we used the structure conservation index (SCI) [45] to assess the quality of the calculated alignments. The SCI is defined as the ratio of consensus folding energy of a set of aligned sequences (calculated using the RNAalifold program [24]) and average unconstrained folding energies of the individual sequences. The SCI is close to 0 for structurally divergent sequences and close to 1 for correctly aligned sequences with a common fold. Values larger than one indicate a perfectly RNA structure which is additionally supported by compensatory as well as consistent mutations that preserve the common structure. The benchmark study [42] established that the SCI is an appropriate measure for RNA alignment quality when the sequences are known to have a common fold, since decreased values of the SCI can be attributed to alignment errors. For the four of the six test sets with reference alignments we also computed the BAliBase SP score (SPS), which directly measure the similarity of two alignments. For all computations we used a fixed tradeoff between sequence and structure scores of *ψ *= 0.5.

**Figure 8:**
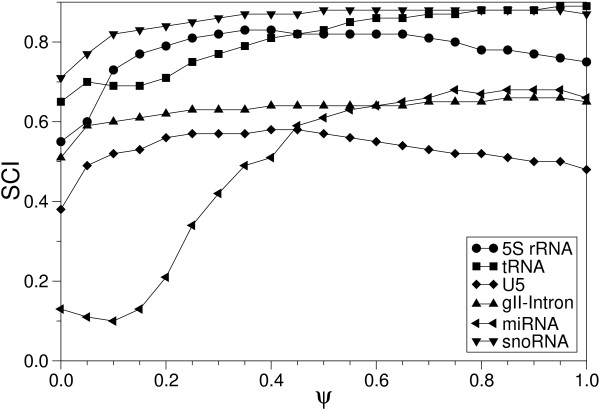
Impact of the balancing parameter *ψ *on SCI mean values for different sets of RNA sequences. *ψ *= 0 fully weights the structure whereas *ψ *= 1 weights the sequence.

We find that aln3nn produces high quality alignments of structured RNAs that are at least competitive with the other methods, including computationally very expensive structure-based methods. In particular, no other program systematically outperforms aln3nn in terms of alignment quality according to either the SCI or the SPS. Interestingly, aln3nn achieves significantly higher SCI values than even the reference alignment on the 5S rRNA data set. Not surprisingly, the performance of structure enhanced alignments depends on the proper weighting of sequence and structure information. Figure 8 shows the influence of the parameter *ψ *on the SCI values for the given RNA sequences. As expected the SCI decreases if structural information is completely ignored (*ψ *= 1). On the other hand, ignoring the sequence information (*ψ *= 0) yields even worse results. The reason is that RNA secondary structure prediction has limited accuracy so that alignments based on predicted structures for individual sequences are based on very noisy data [24,32]. The impact of the *ψ *parameter varies between different RNA families. While alignments of group II introns and U5 spliceosomal RNAs are fairly robust against variations in *ψ*, we observe large variations for miRNA and 5S rRNA alignments.

### 3.4 Gap removals

The possibility to correct gaps that are introduced at early stages was a major motivation for developing aln3nn. We therefore investigated to what extent the algorithm actually utilizes this feature. Table 1 shows the frequency *f *of gaps that are removed at intermediate division steps and that are not re-introduced at later stages. We find that in some data sets one fifth of the gaps in the early stages of the progressive alignment are later removed again. This observation emphasizes the fact that the "once a gap, always a gap" property of pair-wise progressive alignment algorithms is a major shortcoming.

**Table 1:** Mean frequency *f *and standard deviation *σ*_*f *_of correctly removed gap columns from the intermediate alignments after the division process.

RNA family	*f*	*σ*_*f*_
Group II Intron	0.138	0.268
miRNA	0.126	0.210
5S rRNA	0.265	0.279
snoRNA	0.131	0.230
tRNA	0.197	0.305
U5	0.083	0.114

## 4 Discussion

We have presented here a novel progressive alignment tool, aln3nn, that uses exact dynamic programming to construct three-way alignments of sequences and profiles and that uses a three-to-two aggregation procedure in the spirit of Neighbor-Net. A direct comparison of exact three-way alignments with progressive alignments of the same three sequences shows that the progressive approach leads to significantly suboptimal scores. The discrepancy increases with sequence diversity and in/del probability. While incurring significant additional computational costs compared to pair-wise, guide-tree based, approaches, aln3nn achieves competitive alignment accuracies on both protein and nucleic acid data on BAliBASE and BRaliBase benchmark data set. The software furthermore provides an option to compute structure enhanced RNA alignments.

Programs such as clustalw employ a variety of heuristic rules that introduce local modifications of the scoring scheme to (partially) compensate for problematic sections of intermediate alignments. In contrast, aln3nn achieves this encouraging performance without any heuristic modifications of the scoring schemes. This indicates that three-way alignments and the more sophisticated aggregation steps provide a significant advantage of pair-wise methods. In particular, the comparison with the performance of t_coffee shows that the shortcoming of initial pairwise alignments cannot be fully overcome even by utilizing consensus information of a collection of pairwise alignments. In particular, we observe that the three-to-two aggregation step, with its division procedure, removed up to one fifth of the previously introduced gap characters, emphasizing that the inability to correct misplaced gaps is major shortcoming of traditional progressive alignment algorithms.

## 5 Conclusion

In its present implementation, aln3nn demonstrates that progressive alignment schemes can produce competitive high quality alignments even without sophisticated scoring functions. This leaves ample room for future improvements. In particular, one might want to include gap penalties that depend on local sequence context in particular in the intermediate profile alignment steps. The division-step for the three-way alignments could also be modified in several ways. A possible approach would infer a phylogenetic tree that is is then subdivided at the longest or the most central edge. In its present implementation, aln3nn is relatively slow compared to many recent multiple alignment methods, although it typically outperforms some of the standard tools. This lack of performance could be alleviated in the future e.g. by improving the branch and bound approach and by anchoring the alignments at very well conserved regions. Overall, aln3nn shows that progressive alignments are a competitive approach that is worth-while to explore.

## Availability and Requirements

Project name:     aln3nn  

Project homepage: 

Operating system(s):   platform independent in principle, tested for LINUX and other UNIX dialects.  An ANSI C compiler is required.   

Programming language: C  License: GNU GPL.  

Restrictions to use by non-academics: none.  

## 6 Authors' contributions

MK and PFS closely collaborated in design and algorithmic aspects of this study and wrote the manuscript together. MK implemented the software and carried out all benchmark simulations.

## Supplementary Material

Additional file 1 Supplementary figure Click here for file

## References

[B1] The aln3nn software.

[B2] Wang L, Jiang T (1994). On the Complexity of Multiple Sequence Alignment. J Comp Biol.

[B3] Thompson J, Higgins D, Gibson T (1994). CLUSTAL W: improving the sensitivity of progressive multiple sequence alignment through sequence weighting, position-specific gap penalties and weight matrix choice. Nucleic Acids Research.

[B4] Hogeweg P, Hesper B (1984). The alignment of sets of sequences and the construction of phylogenetic trees. An integrated method. J Mol Evol.

[B5] Feng D, Doolittle R (1987). Progressive sequence alignment as a prerequisite to correct phylogenetic trees. J Mol Evol.

[B6] Saitou N, Nei M (1987). The neighbor-joining method: a new method, for reconstructing phylogenetic trees. Mol Biol Evol.

[B7] Sokal RR, Michner CD (1958). A statistical method for evaluating systematic relationships. Univ Kans Sci Bull.

[B8] Bryant D, Moulton V (2002). NeighborNet: An agglomerative method for the construction of planar phylogenetic networks. WABI '02: Proceedings of the Second International Workshop on Algorithms in Bioinformatics.

[B9] Needleman SB, Wunsch CD (1970). A general method applicable to the search for similarities in the aminoacid sequences of two proteins. J Mol Biol.

[B10] Dewey TG (2001). A sequence alignment algorithm with an arbitrary gap penalty function. J Comp Biol.

[B11] Gotoh O (1982). An improved algorithm for matching biological sequences. J Mol Biol.

[B12] Gotoh O (1986). Alignment of three biological sequences with an efficient traceback procedure. J theor Biol.

[B13] Konagurthu A, Whisstock J, Stuckey P (2004). Progressive multiple alignment using sequence triplet optimization and three-residue exchange costs. J Bioinf and Comp Biol.

[B14] Myers E, Miller W (1988). Optimal alignemnts in linear space. Bioinformatics.

[B15] Lipman D, Altschul S, Kececioglu J (1989). A tool for multiple sequence alignment. Proceedings of the National Academy of Sciences of the United States of America.

[B16] Gupta S, Kececioglu J, Schaffer A (1995). Improving the practical space and time efficiency of the shortest-paths approach to sum-of-pairs multiple sequence alignment. Journal of Computational Biology.

[B17] Stoye J (1997). Multiple sequence alignment with the divide-and-conquer method. Gene Combis.

[B18] Bryant D, Moulton V (2004). Neighbor-Net: An Agglomerative Method for the Construction of Phylogenetic Networks. Mol Biol Evol.

[B19] Bryant D, Moulton V (2007). Consistency of Neighbor-Net. Alg Mol Biol.

[B20] Bandelt HJ, Dress AWM (1992). Split Decomposition: A New and Useful Approach to Phylogenetic Analysis of Distance Data. Mol Phyl Evol.

[B21] Huson DH (1998). SplitsTree: analyzing and visualizing evolutionary data. Bioinformatics.

[B22] Wetzel R (1995). Zur Visualisierung abstrakter Ähnlichkeitsbeziehungen. PhD thesis.

[B23] Hofacker I, Fontana W, Stadler P, Bonhoeffer L, Tacker M, Schuster P (1994). Fast folding and comparison of RNA secondary structures. Monatshefte für Chemie.

[B24] Hofacker I, Fekete M, Stadler P (2002). Secondary structure prediction for aligned RNA sequences. J Mol Evol.

[B25] The Vienna RNA package.

[B26] Siebert S, Backofen R (2005). MARNA: multiple alignment and consensus structure prediction of RNAs based on sequence structure comparisons. Bioinformatics.

[B27] Höchsmann M, Töller T, Giegerich R, Kurtz S (2003). Local Similarity in RNA Secondary Structures. Proc of the Computational Systems Bioinformatics Conference, Stanford, CA, August 2003 (CSB 2003).

[B28] Sankoff D (1985). Simultaneous solution of the RNA folding, alignment, and proto-sequence problems. SIAM J Appl Math.

[B29] Hull Havgaard JH, Lyngsø R, Stormo GD, Gorodkin J (2005). Pairwise local structural alignment of RNA sequences with sequence similarity less than 40%. Bioinformatics.

[B30] Mathews DH, Turner DH (2002). Dynalign: An Algorithm for Finding Secondary Structures Common to Two RNA Sequences. J Mol Biol.

[B31] Will S, Missal K, Hofacker IL, Stadler PF, Backofen R (2006). Inferring Non-Coding RNA Families and Classes by Means of Genome-Scale Structure-Based Clustering. PLoS Comp Biol.

[B32] Bonhoeffer LS, McCaskill JS, Stadler PF, Schuster P (1993). RNA Multi-Structure Landscapes. A Study Based on Temperature Dependent Partition Functions. Eur Biophys J.

[B33] Dalli D, Wilm A, Mainz I, Steger G (2006). STRAL: progressive alignment of non-coding RNA using base pairing probability vectors in quadratic time. Bioinformatcs.

[B34] McCaskill J (1990). The equilibrium partition function and base pair binding probabilities for RNA secondary structure. Biopolymers.

[B35] Stoye J, Evers D, Meyer F (1998). Rose: generating sequence families. Bioinformatics.

[B36] Thompson J, Plewniak F, Poch O (1999). BAliBASE: a benchmark alignment databse for the evaluation of multiple alignment programs. Bioinformatcs.

[B37] Do CB, Mahabhashyam MSP, Brudno M, Batzoglou S (2005). PROBCONS: Probabilistic Consistency-based Multiple Sequence Alignment. Genome Research.

[B38] Edgar RC (2004). MUSCLE: a multiple sequence alignment method with reduced time and space complexity. BMC Bioinformatics.

[B39] Notredame C, Higgins D, Heringa J (2000). T-Coffee: A novel method for multiple sequence alignments. Journal of Molecular Biology.

[B40] Katoh K, Misawa K, Kuma K, Miyata T (2002). MAFFT: a novel method for rapid multiple sequence alignment based on fast Fourier transform. Nucleic Acids Res.

[B41] Griffiths-Jones S, Moxon S, Marshall M, Khanna A, Eddy S, Bateman A (2005). Rfam: annotating noc-coding RNAs in complete genomes. Nucleic Acid Research.

[B42] Gardner P, Wilm A, Washietl S (2005). A benchmark of multiple sequence alignment programs upon structural RNAs. Nucleic Acids Research.

[B43] Hertel J, Lindemeyer M, Missal K, Fried C, Tanzer A, Flamm C, Hofacker I, Stadler P (2006). The Expansion of the Metazoan MicroRNA Repertoire. BMC Genomics.

[B44] Hertel J, Hofacker IL, Stadler PF (2007). snoReport: Computational identification of snoRNAs with unknown targets. http://www.bioinf.uni-leipzig.de/Publications/07wplist.html.

[B45] Washietl S, Hofacker I, Stadler P (2005). Fast and reliable prediction of noncoding RNAs. PNAS.

