# Supplementary figures and images for: Progressive multiple sequence alignments from triplets
Source: BMC Bioinformatics. 2007 Jul 15;8:254. doi: 10.1186/1471-2105-8-254 (PMC1948021; doi:10.1186/1471-2105-8-254)

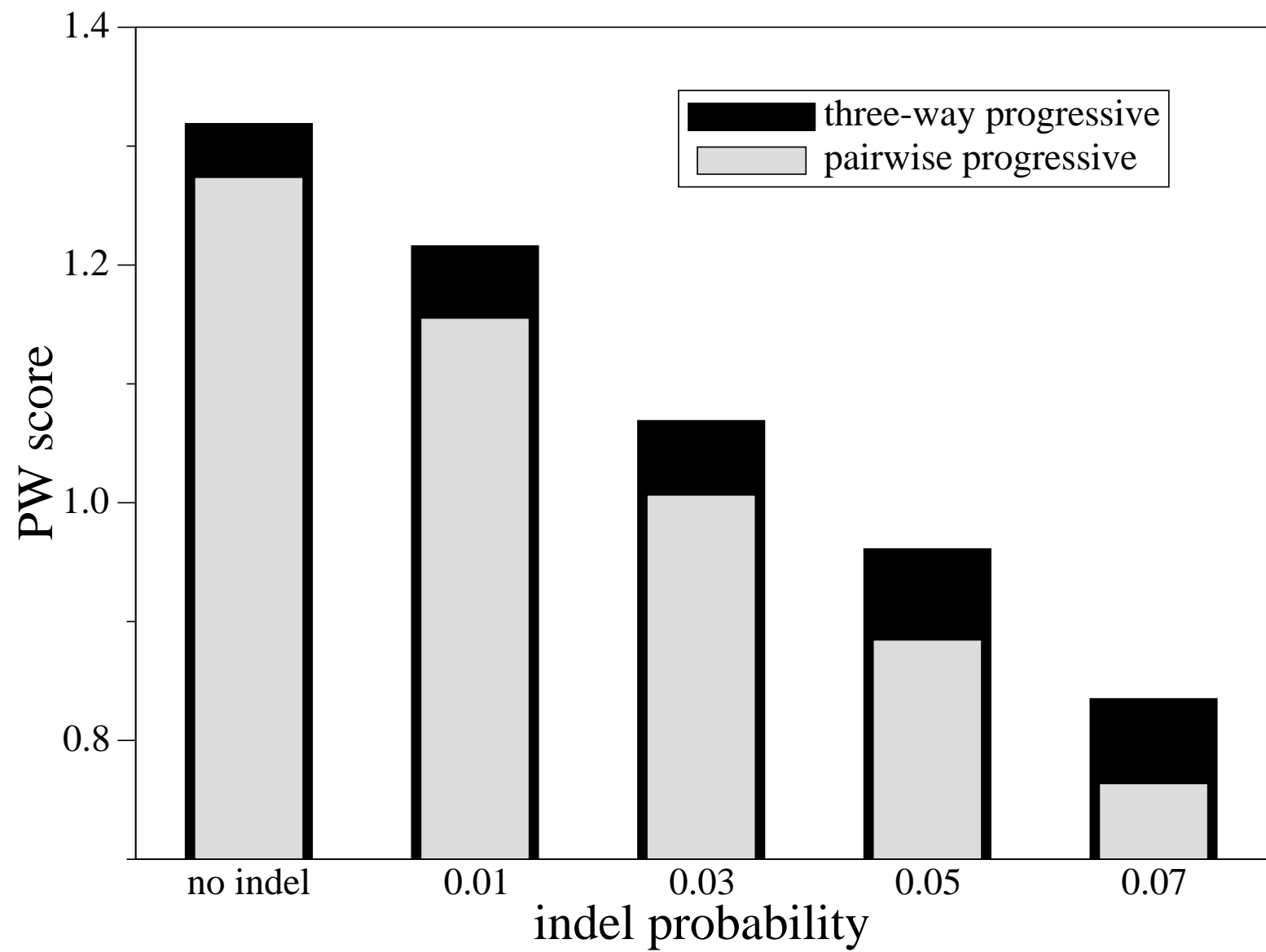

Supplement: Additional file 1 — Supplementary figure [file 1471-2105-8-254-S1.pdf]
